# CGRP in Childhood and Adolescence Migraine: (Patho)physiological and Clinical Aspects

**DOI:** 10.1007/s11916-022-01047-5

**Published:** 2022-03-30

**Authors:** Stefan Evers

**Affiliations:** 1 Department of Neurology, Lindenbrunn Hospital, 31,863 Copppenbrügge, Germany; 2 Faculty of Medicine, University of Münster, Münster, Germany

**Keywords:** Migraine, Calcitonin gene-related peptide (CGRP), Triptans, Gepants, Antibodies

## Abstract

**Purpose of Review:**

To summarise and analyse the current knowledge of CGRP metabolism in childhood and adolescence and its role in childhood and adolescence migraine.

**Recent Findings:**

Influencing CGRP pathways is nowadays one of the main mechanisms to treat migraine. In adults, several clinical trials with different drug classes have supported this finding. However, only very little is known on these mechanisms in children and adolescents with migraine. Based on a literature search, it can be concluded that substantial parts of the CGRP pathways are already developed and working in the preterm fetus of animals. Newborn animals show high CGRP levels and high density of CGRP positive neurons and nerve fibres. In human studies, increased levels of CGRP were observed in childhood and adolescent migraine patients. Remedies based on influencing CGRP metabolism are also working in that age group. For triptans, this has clearly been shown; for gepants, no data are available, and for CGRP ligand/receptor antibodies, positive evidence is only available from case series.

**Summary:**

Only very little is known on CGRP metabolism in childhood and adolescence. However, placebo-controlled clinical trials both on CGRP antagonists and on CGRP ligand/receptor antibodies are under way and will show in some years whether these drug classes are efficacious also in children and adolescents.

## Introduction

Calcitonin gene-related-peptide (CGRP) has become the central molecule and neuropeptide in the basic and clinical research on migraine in the last decades. Since the late 1980s, a lot of knowledge has been accumulated and we understand many although not all mechanisms of CGRP in migraine, and we know several drugs working efficiently against migraine via the CGRP pathway. However, all this research has been performed in adults, and only very little is known about specific mechanisms, pathways, and clinical results in childhood and adolescence migraine. The aim of this review is to give an overview on the role of CGRP in childhood and adolescence if different from adults and on CGRP mechanisms in the treatment of migraine in childhood and adolescence.

Based on a literature search in MedLine and Embase with the search terms “child*”, “adolescen*”, “CGRP”, and “migraine”, findings on the role of CGRP in this context were collected and analysed. Since there is only very little known on the specific aspects of CGRP in childhood and adolescence, this can only be a short narrative review.

## Findings in Animal Models

In 2003, Dong et al. could show that two of the CGRP-A receptor components, CRLR and RAMP1, are expressed in rat placenta [1]. The primary distribution of CRLR and RAMP1 is, among others, in the cytotrophoblast and syncytium in the labyrinth, in trophoblastic giant cells, and in the endothelium and smooth muscle cells of fetal vessels. Before, this group had demonstrated the expression of CGRP-B receptors in rat placenta and its regulation by steroid hormones [2]. In addition to the CGRP-B receptor, the CGRP-A receptors are also present in the rat placenta. Similar to the CGRP-B receptors, levels of CGRP-A receptor components in the placenta are increased with advancing gestation and decreased at term labour. However, the source of CGRP in the feto-placental circulation is not clear. Reports have shown that CGRP concentrations in cord blood are higher than those in the mother’s plasma at term [3]. Therefore, it is possible that CGRP in the fetal circulation is derived from sensory neurons of the fetus himself, or even from the placenta itself. This would mean that the fetus is already able to produce and release CGRP before term.

One of the first studies investigating the developmental changes occurring to peptidergic sensory neurons was performed in the ageing guinea pig. It was discovered that the density of peptidergic neurons surrounding the mesenteric and carotid arteries increase with fetal development, with a peak at birth. The nerve plexuses containing CGRP then decline with age, going down to about half-maximum density once the animal had reached two years of age [4]. Similarly, a study investigating peptidergic innervation of the ageing rat aorta showed that CGRP-positive neuronal fibres were present in animals younger than six months, but gradually disappeared to complete absence when animals reached one year of age [5]. In aged female rats, circulating levels of CGRP have been shown to decline slightly compared with younger animals, and the amount of bioavailable CGRP detected within the mesenteric vascular bed showed an even more profound decline. Supplementation of female sex steroid hormones was able to reverse this reduction in CGRP availability [6].

After capsaicin instillation, adolescent rats showed a comparable trigeminal neuronal activation as adult rats; however, the responsiveness to peripheral trigeminovascular stimulation was lower than in adult rats, including CGRP immunoreactivity in the trigeminal ganglion, protein extravasation, and CGRP depletion in the dura mater [7]. The age-dependent differences in responsiveness to trigeminovascular stimulation in the intracisternal capsaicin-induced migraine model of rats might reflect the lower severity of migraine in pediatric patients.

When examining the distribution of CGRP by indirect immunofluorescence technique, it was found that in the Gasserian ganglion and spinal nucleus of the human trigeminal nerve, CGRP is present, among others, in almost 50% of primary sensory neurons [8]. Morphometric analysis revealed that the CGRP-positive neuronal population is heterogeneous in cell size. The percentage of CGRP-immunoreactive positive cells reached a maximum at perinatal stages, then remained constant, and declined in old age. Pericellular basket-like nerve fibres were detectable only in fetal and pre-term and full-term newborn tissue.

## Findings in Children and Adolescents

CGRP release in migraine attacks of adults was one of the first clinical findings regarding CGRP homoeostasis in migraine [9]. Higher CGRP levels in adult migraine patients compared to healthy control subjects have also been shown between attacks [10].

This has also been shown for children and adolescents [11–13]. In the most recent study, the mean plasma CGRP level in young migraine patients during (291 + / − 60 pg/ml) or between (240 + / − 48 pg/ml) migraine attacks was higher than in other headache patients (51 + / − 5 pg/ml; *p* = 0.006 and 0.018, respectively) and in controls (53 ± 6 pg/ml; *p* = 0.016 and 0.045, respectively). Young patients with high migraine frequency had higher plasma CGRP levels (364 + / − 62 pg/ml) than those with low migraine frequency (183 + / − 54 pg/ml) (*p* = 0.031). It was concluded that the plasma CGRP level can differentiate migraine from non-migraine headache [13]. These results might be relevant for young children who cannot clearly describe their headache symptoms. The results may also provide new insights into the clinical practice for the diagnosis and treatment of pediatric migraine. However, another study could not replicate the finding of increased CGRP levels in pediatric migraine patients both not in the interval and not in the attack [14].

Measurement of salivary CGRP has been proposed as a non-invasive biomarker in healthy subjects [15] and in migraine patients [16]. The possibility of measuring CGRP in salivary samples has also been shown in children and adolescents [17]. However, there was no clear correlation between blood and salivary CGRP level. Following a complementary intervention (forced breathing), children showed a sympathetic output leading to a decrease of salivary CGRP, of heart rate, and of systolic blood pressure.

Triptans probably work via blocking release of CGRP. This has been suggested by clinical studies in adults [18]. If triptans work also in children, this would therefore be an argument that CGRP metabolism does also play a role in childhood and adolescence migraine pathophysiology. The first clinical studies on the efficacy of triptans in childhood were negative [19]. Therefore, it was believed that the CGRP metabolism does not play a major role in childhood migraine. However, it was also found that a high placebo response in children and adolescents might be responsible for the first negative placebo-controlled clinical triptan-trials on migraine in children and adolescents. Later studies, however, after adapting the study design to the high placebo response, could show a significant efficacy of triptans also in children and adolescence [20]. This supports the finding that CGRP pathways are highly developed in childhood and adolescence and that these pathways might be important for migraine in that age group. Lasmiditan, a novel antimigraine drug also blocking central CGRP release [21], has been shown to be safe and well tolerated in a small sample of adolescents with migraine [22].

No published data on children and adolescents could be found for the gepants which have recently been developed as small molecule CGRP antagonist against migraine. However, studies are registered in clinical trial databases (see Table 1) and it might be in some years that we will see data on the use of gepants in children and adolescents.

**Table 1:** Planned or recruiting studies on migraine treatment with a CGRP antagonist or a CGRP ligand/receptor antibody in the EudraCT and in the ClinicalTrials.gov databases (some trials are registered in both databases). Last access December 23, 2021

EudraCT number:	Title of the trial:
2018–000923-15	Full Title: A phase I, randomized, open-label, multiple-dose study to evaluate safety, tolerability, and pharmacokinetics of AMG 334 in children and adolescents with migraine
2020–003517-35	Full Title: Phase 3, multicenter, randomized, double-blind, group sequential, placebo-controlled study to assess efficacy and safety of rimegepant for the treatment of migraine (with or without aura) in children
2017–002397-39	A phase 3, Randomized, double-blind, placebo-controlled, parallel-group study to evaluate the efficacy and safety of erenumab in children (6 to < 12 years) and adolescents (12 to < 18 years) with episodic migraine
2017–002399-23	A phase 3, randomized, double-blind, placebo-controlled, parallel-group study to evaluate the efficacy and safety of erenumab in children (6 to < 12 years) and adolescents (12 to < 18 years) with chronic migraine
2018–004622-28	A randomized, double-blind, placebo-controlled study of galcanezumab in adolescent patients 12 to 17 years of age with chronic migraine – the REBUILD-2 study
2018–000734-35	A single-dose, open-label study to characterize the pharmacokinetics, safety and tolerability of subcutaneous administration of fremanezumab in pediatric migraine patients (6 to 11 years of age …
2019–002056-16	A multicenter, open-label study evaluating the long-term safety, tolerability, and efficacy of monthly subcutaneous administration of fremanezumab for the preventive treatment of episodic and chron
ClinicalTrials.gov number:	Title of the trial:
NCT05127954	Long-term extension study to assess safety and tolerability of oral ubrogepant tablets for the acute treatment of migraine in children and adolescents (ages 6–17)
NCT04537429	A study in children and young people with migraine to learn what the body does to eptinezumab
NCT05125302	Study to assess adverse events and disease activity of oral ubrogepant tablets for the acute treatment of migraine in children and adolescents (ages 6–17)
NCT04743141	Long-term safety study of rimegepant in pediatric subjects for the acute treatment of migraine
NCT05164172	A study with eptinezumab in children and adolescents (6 to 17 years) with chronic or episodic migraine
NCT05156398	Efficacy and safety study of rimegepant for the preventative treatment of migraine in pediatric subjects
NCT03832998	Efficacy and safety of erenumab in pediatric subjects with chronic migraine (OASIS (CM))
NCT03836040	Efficacy and safety of erenumab in pediatric subjects with episodic migraine (OASIS (EM))
NCT04965675	A study with eptinezumab in adolescents (12–17 years) with chronic migraine (PROSPECT-2)
NCT04530110	A study to test if fremanezumab is effective in preventing migraine in children and adolescents
NCT03499119	AMG 334 20,160,172 pediatric migraine PK study
NCT04616326	A study of galcanezumab (LY2951742) in participants 12 to 17 years of age with chronic migraine (REBUILD-2)
NCT03432286	A study of galcanezumab (LY2951742) in participants 6 to 17 years of age with episodic migraine (REBUILD-1)
NCT04458857	A study to test if fremanezumab is effective in preventing episodic migraine in patients 6 to 17 years of age
NCT04464707	A study to test if fremanezumab is effective in preventing chronic migraine in patients 6 to 17 years of age

In addition, it has to be noted that no data are available on the effect of CGRP or of CGRP modifying drugs on puberty. Also, accompanying symptoms such as photophobia and phonophobia are probably not related to CGRP. This aspect is important since children and adolescents show often lower severity of these symptoms.

## CGRP Ligand/Receptor Antibodies in Children and Adolescents

The four CGRP ligand/receptor antibodies which have been developed for the treatment of migraine in adults have not been studied and therefore not been approved for the use of patients under the age of 18. Clinical trials for that age group are under way (see Table 1), but no placebo-controlled trial has been published yet.

In preparation of such clinical trials, the dosage of the CGRP ligand/receptor antibodies in children and adolescents had to be determined. Pediatric pharmacokinetic studies of the antibodies have been finished, are planned, or are ongoing. In a phase 1 study on fremanezumab, children 6 to 11 years of age and weighing 17 to 45 kg received a single subcutaneous dose of 75 mg [23••]. The pharmacokinetic data were then used to refine the adult model and create concentration–time profiles in a virtual pediatric population resulting in an appropriate subcutaneous injection dose of fremanezumab 120 mg monthly in pediatric patients weighing less than 45 kg.

As reported above, data from placebo-controlled trials on children and adolescents are not available. In such trials, it would be important to use also age-specific patient related outcome measures such as the pedMIDAS.

However, in an open multicentre retrospective chart review, 112 adolescents who had received at least one dose of a CGRP ligand/receptor antibody were analysed [24]. Mean age at first dose was 15.9 years. They had chronic migraine (*n* = 94), new daily persistent headache (*n* = 12), and persistent post-traumatic headache (*n* = 6). At first follow-up visit, there was a significant reduction in headache frequency compared with baseline by − 2.0 days. Significant benefit with respect to headache days and to functional improvement was reached by 22% to 30% within two follow-ups. Side effects were similar to those seen in adults (injection site reaction, constipation). Another small case series reported significant improvement of migraine in 50% of adolescent patients by erenumab [25••].

In order to prepare the use of CGRP ligand/receptor antibodies in childhood and adolescence migraine, a US American manuscript written by members of the Pediatric & Adolescent Headache special interest group of the American Headache Society was published and meant to serve as expert opinion [26]. It reported recommendations on the use of CGRP ligand/receptor antibodies in children and adolescents. This consortium did not completely deny the use of these antibodies in this age group although they are not approved, but they gave the recommendations listed in Table 2. Interestingly, the use recommendations were not restricted to migraine but also other chronic headache disorder such as cluster headache, new daily persistent headache (NDPH), and persistent headache attributed to traumatic injury to the head were considered.

**Table 2:** Recommendations of a special interest group of the American Headache Society for the use of CGRP ligand/receptor antibodies in children and adolescents [26]

**Indications**
≥ 8 headache days per month
PedMIDAS score ≥ 30
Failure of ≥ 2 preventive therapies (pharmacologic, nutraceutical, and/or non-pharmacologic)
Post-pubertal adolescent, or pre-pubertal child in selected cases
**Contraindications**
Disturbed blood–brain barrier (e. g. recent history of meningitis, recent neurosurgery)
Severe cardiovascular disease, stroke
Pregnancy, planned pregnancy, or breast-feeding
**Monitoring**
Pubertal status
Bone health, consider checking vitamin D status
Linear growth
Weight/BMI
Infections
Pregnancy status

The authors of this paper conclude that, until studies in children and adolescents are completed, CGRP ligand/receptor antibodies should be considered “primarily for post-pubertal adolescents experiencing relatively frequent migraine (i. e. ≥ 8 headache days per month) with moderate or severe migraine-related disability (as measured by PedMIDAS or other validated instruments). In the event that a younger child has severe chronic migraine that has proven refractory to multiple migraine preventive trials, it would be reasonable to consider” CGRP ligand/receptor antibodies with a careful monitoring.

## Conclusions

Based on this short review, it can be concluded that substantial parts of the CGRP pathways are already developed and working in the preterm fetus. Young animals show a high CGRP level and a high density of CGRP positive neurons and nerve fibres. This is also probably true for children and adolescents. In human studies, increased levels of CGRP were observed in childhood and adolescent migraine patients. Therefore, it can be assumed that remedies based on influencing CGRP metabolism are also working in that age group. Furthermore, it is not unlikely that children and adolescents need higher body weight adjusted doses of anti-CGRP drugs compared to adults.

For triptans, it has already been shown that they work in younger ages, for gepants, no data are available, and for CGRP ligand/receptor antibodies, positive evidence is only available from case series. However, placebo-controlled clinical trials both on CGRP antagonists and on CGRP ligand/receptor antibodies are under way and will show in some years whether these drug classes are efficacious also in children and adolescents. Until that time, children and adolescents should be treated with gepants or with CGRP ligand/receptor antibodies only in clinical trials or if they suffer from very severe migraine and are refractory to all conventional migraine prophylactic drugs.
